# Impact of Polyphenol Antioxidants on Cycling Performance and Cardiovascular Function

**DOI:** 10.3390/nu6031273

**Published:** 2014-03-24

**Authors:** Joel D. Trinity, Matthew D. Pahnke, Justin R. Trombold, Edward F. Coyle

**Affiliations:** Human Performance Laboratory, Department of Kinesiology and Health Education, The University of Texas, Austin, TX 78712, USA; E-Mails: joel.trinity@utah.edu (J.D.T.); matthew.pahnke@pepsico.com (M.D.P.); justin.trombold@utsouthwestern.edu (J.R.T.)

**Keywords:** thermoregulation, exercise, antioxidant

## Abstract

This investigation sought to determine if supplementation with polyphenol antioxidant (PA) improves exercise performance in the heat (31.5 °C, 55% RH) by altering the cardiovascular and thermoregulatory responses to exercise. Twelve endurance trained athletes ingested PA or placebo (PLAC) for 7 days. Consecutive days of exercise testing were performed at the end of the supplementation periods. Cardiovascular and thermoregulatory measures were made during exercise. Performance, as measured by a 10 min time trial (TT) following 50 min of moderate intensity cycling, was not different between treatments (PLAC: 292 ± 33 W and PA: 279 ± 38 W, *p* = 0.12). Gross efficiency, blood lactate, maximal neuromuscular power, and ratings of perceived exertion were also not different between treatments. Similarly, performance on the second day of testing, as assessed by time to fatigue at maximal oxygen consumption, was not different between treatments (PLAC; 377 ± 117 s *vs*. PA; 364 ± 128 s, *p* = 0.61). Cardiovascular and thermoregulatory responses to exercise were not different between treatments on either day of exercise testing. Polyphenol antioxidant supplementation had no impact on exercise performance and did not alter the cardiovascular or thermoregulatory responses to exercise in the heat.

## 1. Introduction

High intensity exhaustive exercise is associated with an excessive elevation in free radical production and subsequent increase in oxidative stress [1,2,3]. Heat stress during exercise has also been shown to exacerbate the increase in oxidative stress [4,5]. Therefore, the combination of exhaustive exercise and heat stress yields an ideal condition to test if antioxidant supplementation can attenuate the deleterious impact of oxidative stress thus improving exercise performance. To date, the results from studies using antioxidants are equivocal as increases [6,7,8,9,10,11,12], no change [13,14,15], and decreases in performance [16] have been reported.

Rather compelling evidence for the beneficial role of antioxidant supplementation and exercise performance has been shown with intravenous infusion of the free radical scavenger *n*-acetylcysteine (NAC) [8,9,10,11,12,17,18,19]. The mechanism by which NAC improves exercise performance is believed to occur through a direct effect of the antioxidant on muscle fiber contractility and fatigability [20]. However, these studies used supra-physiological dosages of NAC that were administered via intravenous infusion. Contrary to these findings, most studies using oral administration of the polyphenol antioxidant, quercetin, have failed to find a beneficial effect on exercise performance [13,14,15]. Only one study, to our knowledge, has shown improved exercise performance with oral quercetin ingestion in humans [7]. Further investigation is required to determine if physiological dosages of antioxidants increase performance in healthy individuals.

The antioxidant supplement used for this study contained a mixture of high potency polyphenol antioxidants (PA), primarily in the form of ellagitannins (punicalagin and ellagic acid), found in pomegranate juice [21]. Polyphenols represent the most abundant dietary source of antioxidants [22] and are part of an emerging field of nutraceuticals based on their biological activity and potent treatment effects in clinical conditions associated with oxidative stress and inflammation including cardiovascular disease [23], type 2 diabetes [24], atherosclerosis [25], cancer [26], and rheumatoid arthritis [27]. However, the ergogenic potential of PA is largely unknown. Previous work has shown PA in pomegranates to possess higher antioxidant potency when compared to other polyphenol rich fruits and pharmacokinetic analysis revealed that plasma levels of the active antioxidants peaked 1 to 2 h after consumption and were present in the urine for 48 h [28,29,30]. PA has been shown to increase nitric oxide (NO) bioavailability by protecting the molecule from oxidative destruction [31]. Given the important roles of NO in cutaneous blood flow [32], thermoregulatory control of sweating [33], skeletal muscle blood flow [34,35], and skeletal muscle contractile efficiency [36,37,38,39] PA derived from pomegranates is an ideal supplement to test for ergogenic properties during exercise in a hot environment.

The purpose of this study was to determine if PA supplementation can improve exercise performance during prolonged exhaustive exercise in the heat and during short duration high intensity fatiguing exercise. Furthermore, we sought to assess potential mechanisms by which improvements in exercise performance may be improved including; cardiovascular strain as measured by central hemodynamics (heart rate [HR], stroke volume [SV], and cardiac output [CO]), thermoregulation, and muscle efficiency. We hypothesized that PA would increase performance during exercise in the heat by improving thermoregulation and muscle efficiency and subsequently reducing cardiovascular strain.

## 2. Experimental Section

### 2.1. Subjects

Twelve healthy and well-trained male cyclists (26.8 ± 5.0 years of age) provided written informed consent to participate in this study. The protocol, experimental design, and informed consent were approved by the Institutional Review Board at The University of Texas at Austin. The subjects’ stature, body mass, and maximal oxygen consumption (VO_2_max) (means ± SD) were as follows: 1.80 ± 0.08 m, 74.4 ± 8.8 kg, 4.47 ± 0.31 L/min, respectively.

### 2.2. Experimental Design

A double blind placebo controlled randomized cross over design was utilized to test the impact of PLAC or PA supplementation on exercise performance during prolonged exhaustive exercise and during high intensity fatiguing exercise. Moreover, indices related to performance (gross cycling efficiency, blood lactate, maximal neuromuscular power, ratings of perceived exertion), cardiovascular strain (HR, SV, CO) and thermoregulatory function (core temperature [Tcore], skin temperature [Tskin], and skin blood flow [SkBF]) were assessed. Experimental trials were performed back-to-back on the final days of each supplementation period. For the remainder of the manuscript these two testing days will be referred to as day 1 and day 2 of the experimental protocol. In order to match diet from one treatment to the next, subjects kept dietary logs for final 3 days of the supplementation period and repeated this intake during the second supplementation period. An overnight fast of at least 10 h was performed prior to the experimental trails to avoid the impact of acute and variable nutrient intake on exercise performance. All testing was performed at the same time of day and subjects maintained their normal training regimen during study enrollment.

**Figure 1:**
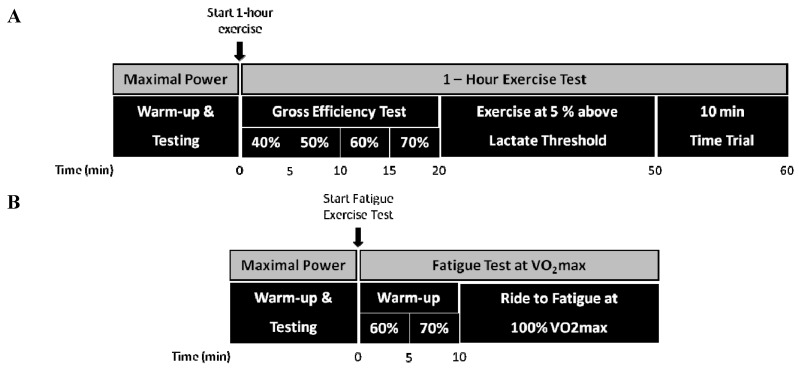
Experimental protocols for prolonged exhaustive exercise in the heat—day 1 (**A**) and exercise to fatigue at VO_2_max—day 2 (**B**). The experimental protocols were performed on consecutive days coinciding with the final two days of the supplementation periods. Prior to the exercise tests subjects performed a standard warm-up and maximal power was assessed. Please refer to the text for a detailed description of each protocol.

Prior to the start of the experimental trials and supplementation intervention subjects reported to the laboratory on three separate occasions to perform preliminary testing. Preliminary testing was completed 7 days prior to commencement of the experimental trials. The purpose of these trials was two-fold: (1) familiarize subjects with the testing procedures and the demands of the exercise tests; and (2) determine the appropriate power outputs for each of the exercise tests. The first preliminary visit included a submaximal and maximal exercise test on a cycle ergometer (Excalibur Sport, Lode, Groningen, The Netherlands). The submaximal test included 5, 5-min stages during which power output was progressively increased by 20 to 40 watts per stage. Lactate samples were collected during the final min of each stage by a finger stick and analyzed with a portable lactate meter (Lactate Pro, Arkray Inc., Kyoto, Japan). Lactate threshold (LT) was determined at the exercise intensity and workrate corresponding to 1 mmol increase above baseline values. Oxygen consumption (VO_2_) was collected continuously during this test and averaged over the final min of each stage to determine the oxygen of cost of cycling at each power output. Following the submaximal test subjects rested for 10 min before performing an incremental VO_2_max test, which lasted 8 to 12 min. Initial power output for the VO_2_max test was set at a power output that elicited approximately 80% of subject’s reported maximal heart rate (HRmax). This first preliminary visit was performed in temperate environmental conditions (24 °C and 50% relative humidity (RH)). The 2nd and 3rd preliminary visits were performed on back-to-back days and mimicked the experimental trials (*i.e*., day 1 and day 2, experimental timeline presented in Figure 1A,B) and are described in detail in the following section.

Upon arrival at the laboratory on day 1 (Figure 1A) subjects were asked to void their bladder and obtain a nude body mass. Subjects then inserted a rectal temperature probe (model 401, Measurement Specialties, Hampton, VA, USA) 12 cm past the anal sphincter and were escorted to the environmental chamber and asked to sit quietly on the ergometer while baseline measures of blood pressure were made (STBP-680, Colins, San Antonio, TX, USA). Subjects then performed a 5 min warm-up at 60% of their VO_2_max. Immediately following the warm-up, subjects exited the environmental chamber and performed 4 maximal power sprint tests on an inertial load ergometer [40]. After the maximal power tests subjects re-entered the environmental chamber and began the 1 h bout of exercise. During the first 20 min of the 1 h test subjects cycled at 40%, 50%, 60%, and 70% of VO_2_max for 5 min for the determination of gross cycling efficiency [41]. At min 20 power output was adjusted to 5% above LT and at min 50 a 10 min TT began. The TT began at a power output that would elicit approximately 90% of VO_2_max. After the first 2 min of the TT the subject was able to change the power output every 30 s. The power output was changed by a member of the research team as instructed by the subject; however, subjects were unaware of the actual power output and time remaining during the TT. Verbal encouragement was given to the subject by a designated member of the research team. The goal of the TT was to perform as much work as possible during the 10 min period. Following the TT subjects obtained a nude body mass. To offset dehydration subjects consumed 1 L of water during the course of the trial. A similar protocol has been successfully used by our lab to assess performance in response to nutritional interventions and reported that the work rate eliciting a VO_2_max of 5% above LT was the highest possible that could be sustained for 1 h in a warm environment when subjects did not consume fluid [42].

On day 2 of the experimental testing (Figure 1B) subjects performed the same warm-up and series of maximal power tests as day 1. After completion of the warm-up and the maximal power tests subjects entered the environmental chamber and began a 10 min exercise bout consisting of 5 min of cycling at 60% and 70% of VO_2_max. At min 10 an open-ended ride to fatigue began. Power output for this performance test was set at the minimal power output needed to elicit 100% of VO_2_max [43]. Previous work from our lab has shown that well trained endurance athletes were able to cycle for 5 to 9 min at this power output [44]. Subjects were verbally encouraged to give a maximal effort during all trials. No fluid was given during this trial due to the short duration. All trials, except Session 1 (preliminary submaximal and VO_2_max tests), were performed in the environmental chamber in warm conditions with fan cooling (dry bulb temperature: 31.5 °C, RH: 55.1%; fan speed: 4 m/s).

### 2.3. Supplementation

Supplements of PA or PLA (500 mL) were taken twice daily at 12 h intervals over the seven-day testing period. On day 6 (day 1 of testing) and day 7 (day 2 of testing), subjects consumed the supplement approximately 30 min before the start of the exercise test. The timing of PA supplementation was based on previous pharmacokinetic analysis revealing peak PA levels in the blood at 1 to 2 h post consumption [29,30], thus the majority of the exercise testing described above corresponded to this timeframe. PA and PLA drinks were provided by POM Wonderful, LLC (Los Angeles, CA, USA). Products were shipped frozen and were stored at 4 °C upon arrival. Each bottle of PA contained 1800-ppm polyphenols, comprised of 95.5% ellagitannins (22.5% as punicalins and punicaligans), 3.5% ellagic acid, and 1% anthocyanins. Both PA and PLA contained a very low amount of carbohydrate (4 g maltodextrin and sucralose) with additional coloring and flavoring to blind the treatments. Subjects were reminded verbally and through e-mail communication to consume the experimental supplements at the required times.

### 2.4. Respiratory Measurements

VO_2_ was determined using a commercially available metabolic cart (Max II Modular Metabolic System, AEI Technologies, Pittsburg, PA, USA) while subjects cycled on an electromagnetically braked ergometer (Excalibur Sport, Lode, Groningen, The Netherlands). Gas analysis was performed using oxygen and carbon dioxide analyzers (Models S-3A/I and CD-3A, respectively, AEI Technologies, Pittsburgh, PA, USA) while the subjects breathed through a one-way valve (Hans Rudolph, Kansas City, MO, USA). Ventilation was measured with an inspiratory pneumotachometer (Hans Rudolph, Kansas City, MO, USA).

### 2.5. Cardiovascular Measurements

An impedance cardiography device (Physioflow Type PF05L1, Manatec Biomedical, Macheren, France) was used to measure HR, SV, and CO. The Physioflow unit uses changes in transthoracic impedance (dZ) in response to an administered electrical current during cardiac ejection to calculate SV. The Physioflow emits a high frequency (75 kHz) and very low-amperage (3.8 mA peak-to-peak) alternating electrical current via skin electrodes (Series 810 electrodes, S & W Healthcare, Brooksville, FL, USA). Two pairs of electrodes, one transmitting and the other receiving, were applied above one another so as to not overlap at the supra-clavicular fossa at the left base of the neck and at the midpoint of the thoracic region of the spine. An additional pair of electrodes was used to monitor a single ECG lead (V1/V6 position). The Physioflow impedance cardiograph has been validated against the direct Fick method [45] and mean differences between CO as measured by the direct Fick and the Physioflow were not found to be significantly different at rest, during submaximal exercise, or during incremental maximal exercise [46]. Prior to placement of electrodes, the skin was cleaned with isopropyl alcohol and a gauze sponge. Subjects wore a Spandage^®^ shirt (Medi-Tech, Brooklyn, NY, USA) around their torso and Coban^®^ tape (3M, Saint Paul, MN, USA) around their neck to reduce movement of electrode wires and to insure that electrodes were kept in place throughout the duration of the exercise tests.

Blood pressure (systolic blood pressure (SBP) and diastolic blood pressure (DBP)) was collected using an automated blood pressure device (Colin STBP-680, Mediana, Redwood, WA, USA). Resting measurements were made while the subject sat quietly on the cycle ergometer. During the 1 h exercise bout on day 1 blood pressure was measured with the subject’s arm relaxed on the handlebars during the final min of each submaximal stage, at min 30 and 45, and again at two min intervals during the 10 min TT. Mean arterial pressure (MAP) was calculated as DBP + 1/3(SBP-DBP).

### 2.6. Body Temperatures and Rating of Perceived Exertion

Core temperature (Tcore) was measured using a rectal temperature probe. Skin temperature (Tskin) was measured using skin thermistors (model 409A, Measurement Specialties, Hampton, VA, USA) at six sites; back, chest, bicep, forearm, thigh and calf and mean Tskin was calculated based on the equation of Hardy and Dubios [47]. All temperature data was collected continuously on a personal computer using Tracer-Daq software (Measurement Computing, Norton, MA, USA) interfaced with an A/D board (USB Temp, Measurement Computing, Norton, MA, USA). Ratings of perceived exertion (RPE) were collected using the Borg 6 to 20 point scale [48] at min 30 and 45 of the one hour exercise bout on day 1.

Skin blood flow (SkBF) requirements were calculated according to the following equation:

SkBF = ((HP/(Trec − Tskin)) − K_o_)/SH (1)

at min 5, 10, 15, 20, and 50 of exercise on day 1; where HP is heat production in W, K_o_ is the thermal conductance of the tissue (14 W·C^−1^), and SH is the specific heat of blood (3.85 kJ·L^−1^·°C^−1^) [49].

### 2.7. Maximal Neuromuscular Power

Maximal neuromuscular power (Pmax) was determined by inertial load ergometry. Rationale for the inclusion and assessment of Pmax is based on previous work reporting improvements in performance and Pmax during a taper (a period of reduced training load designed to improve recovery and exercise performance) [50,51]. Therefore, Pmax was assessed during the PLAC and PA supplementation periods to assess possible improvements in recovery and training status. Validation and reliability of the inertial-load method has been previously described in detail [40]. Briefly, the inertial-load ergometer uses the resistance created by the moment of inertia of a flywheel to represent the force that the subject accelerates during the test. Power is calculated as the product of inertia, angular velocity and angular acceleration. Flywheel angular velocity and acceleration are determined by an optical sensor and micro-controller based computer interface which measures time (±0.5 ms) and allows power to be calculated instantaneously and averaged over one complete revolution of the pedal cranks (Pmax). The coefficient of variation with this technique is ±2.5% and this high degree of reliability has allowed mean treatment differences in maximal power of 2%–3% within a population to become statistically significant [50,51]. The measurement of Pmax requires maximal effort over 3 to 4 s with 60 s of passive recovery between efforts. Subjects performed familiarization with the inertial load ergometer during preliminary visit. Four measures of Pmax were collected during day 1 and day 2 of the experimental trials. All Pmax values were obtained after a 5 min warm-up and prior to the prolonged exercise tests.

### 2.8. Statistics

All statistical analyses were performed using SPSS version 14.0 (IBM, Armonk, NY, USA). Data are presented as mean ± standard deviation of the mean. For the purpose of clarity, data in the figures are presented as mean ± standard error. A two-way (trial *x* time) repeated measures ANOVA was used to test for significant differences. *A priori* analyses of sequential time points were employed following a significant main effect of time. The number of *a priori* comparisons was limited to *k* − 1 for each variable measured, where *k* is equal to the number of means compared. If the interaction (treatment *x* time) was found to be significant, pairwise comparisons were made using Fisher’s least significant difference test. If Mauchly’s test of sphericity was violated, the Greenhouse-Geisser correction was used to correct for this violation. Average power during the TT (day 1) and time to fatigue (day 2) were analyzed using a paired samples T-test. For statistical analysis, data from day 1 were separated into two groups; data from rest to 50 min of exercise and data during the 10 min TT. Significance was accepted at the *P* < 0.05 level.

## 3. Results

### 3.1. Subject Characteristics and Environmental Factors

Body mass prior to (PLAC; 74.2 ± 9.1 and PA; 74.4 ± 9.1 kg, *p* = 0.32) and after (PLAC; 73.7 ± 8.8 and PA; 73.9 ± 9.1 kg, *p* = 0.16) the 1 h exercise bout on day 1 was not different between treatments

### 3.2. Exercise Intensity and Respiratory Responses

**Figure 2:**
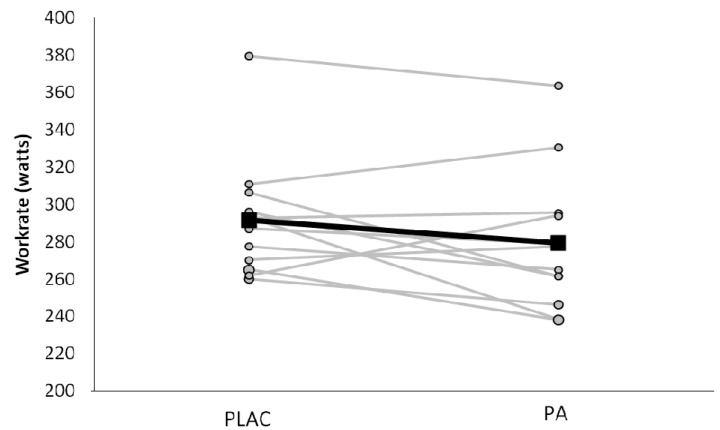
Average power output (watts) during the 10 min time trial performed on day 1, placebo (PLAC) and polyphenol antioxidant (PA) trials. The 10 min time trial was performed following 50 min of moderate intensity (5% above the lactate threshold) cycling exercise in the heat (31.5 ± 0.7 °C, 55% ± 4% relative humidity). Individual data of the 12 subjects and the mean response, represented by solid black line. There was no difference in average power output between trials, *p* = 0.12.

During the first 20 min of exercise on day 1 subjects cycled at power outputs of 99 ± 11, 144 ± 12, 189 ± 15, and 234 ± 19 W which elicited 42% ± 4% , 53% ± 4%, 63% ± 4% , and 74% ± 5% of VO_2_max. Respiratory exchange ratio (RER) and gross efficiency (GE) during cycling was not influenced by the PA supplementation (Table 1). Average power output during the 10 min TT (day 1) was not different between treatments (PLAC; 292 ± 33 and PA; 279 ± 38 W; *p* = 0.12) (Figure 2). Lactate levels at min 5 (PLAC; 1.3 ± 0.3 and PA 1.4 ± 0.4), min 30 (PLAC; 2.6 ± 0.9 and PA; 2.7 ± 0.9), and post TT (PLAC; 8.2 ± 2.4 and PA 8.1 ± 3.3) were not different between trials (all *p* > 0.05). Similarly, RPE was not different between trials at min 30 (PLAC; 13.3 ± 1.2 and PA; 13.3 ± 0.9) and 45 (PLAC; 13.5 ± 1.3 and PA; 14.1 ± 1.3) (both *p* > 0.05).

**Table 1:** Respiratory responses and gross cycling efficiency. Values are mean ± SD of 12 subjects.

Time, min	5	10	15	20
**VO_2_, mL/min**				
**PLAC**	1807 ± 147	2347 ± 240 *	2760 ± 218 *	3271 ± 224 *
**PA**	1882 ± 171	2354 ± 192 *	2839 ± 235 *	3324 ± 284 *
**RER**				
**PLAC**	0.84 ± 0.03	0.87 ± 0.03 *	0.87 ± 0.03	0.88 ± 0.04 *
**PA**	0.84 ± 0.03	0.86 ± 0.03 *	0.87 ± 0.03 *	0.89 ± 0.04 *
**GE, %**				
**PLAC**	16.2 ± 1.5	18.1 ± 1.4 *	20.1 ± 1.0 *	20.9 ± 1.2 *
**PA**	15.6 ± 1.5	18.1 ± 1.3 *	19.6 ± 1.1 *	20.6 ± 0.9 *

Placebo (PLAC), polyphenol antioxidant (PA), Oxygen consumption (VO_2_), respiratory exchange ratio (RER), gross efficiency (GE). The power output elicited 40% of VO_2_max from min 0–5, 50% of VO_2_max from min 5–10, 60% of VO_2_max from min 10–15, and 70% of VO_2_max from min 15–10. * Indicates significant difference from previous value, *p* < 0.05.

**Figure 3:**
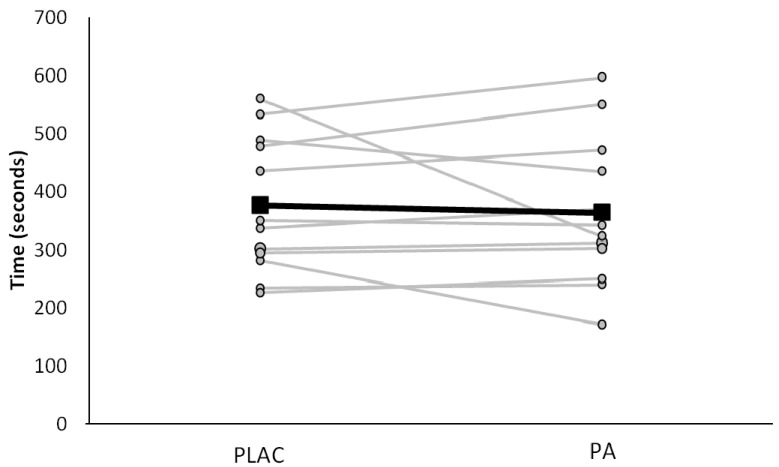
Time to fatigue (s) during the constant load performance test on day 2, placebo (PLAC) and polyphenol antioxidant (PA). Subjects cycled until volitional exhaustion in the heat (31.5 ± 0.7 °C, 55% ± 4% relative humidity) at the power output (338 ± 27 watts) required to elicit maximal oxygen consumption. Individual data of the 12 subjects and the mean response, are represented by solid black line. There was no significant difference in time to fatigue between PLA and PA, *p* = 0.61.

During the fatigue test (day 2) subjects cycled at a constant power output of 338 ± 27 W. There were no significant differences in time to fatigue between the two treatments (PLAC; 377 ± 117 s *vs*. PA; 364 ± 128 s, *p* = 0.61) (Figure 3).

Measures of maximal power (Pmax), instantaneous power (IP), and velocity at maximal power (RPM) were not different between day 1 and day 2 or between treatments (Table 2).

**Table 2:** Maximal neuromuscular power. Values are mean ± SD of 12 subjects.

Parameters	Placebo (Day 1)	PA (Day 1)	Placebo (Day 2)	PA (Day 2)
Pmax, watts	1250 ± 231	1240 ± 229	1240 ± 214	1243 ± 258
IP, watts	2027 ± 384	2029 ± 362	2027 ± 336	2046 ± 415
RPM	121 ± 9	121 ± 9	120 ± 9	117 ± 8
Pmax, W/kg	16.8 ± 1.8	16.6 ± 1.9	16.7 ± 1.6	16.6 ± 2.0

Polyphenol antioxidant (PA), maximal neuromuscular power (Pmax), instantaneous maximal power (IP), revolutions per minute at maximal power (RPM), and maximal power per kilogram body mass (Pmax, W/kg). No significant differences between treatments or over time were observed.

### 3.3. Cardiovascular Responses

**Figure 4:**
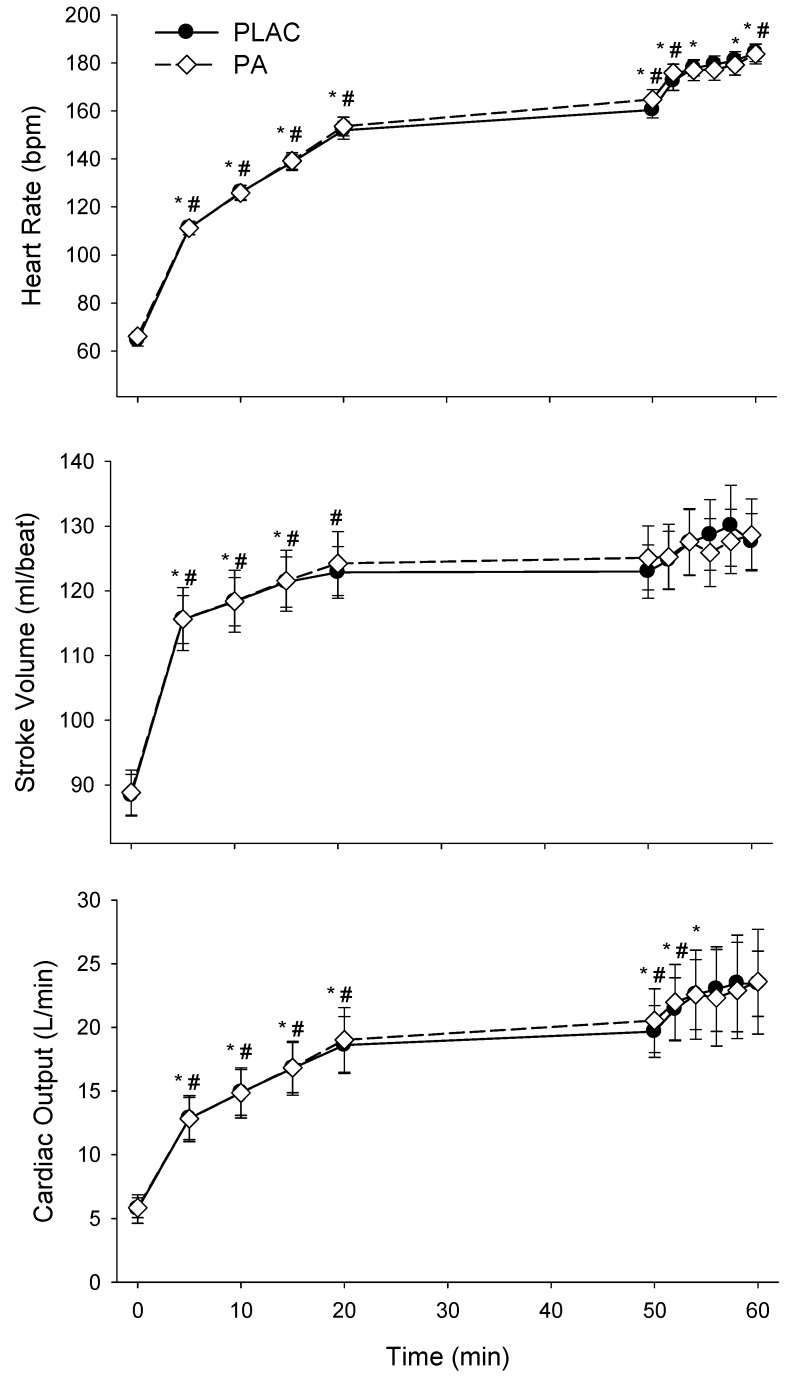
Central hemodynamic responses to exercise, day 1, placebo (PLAC, •) and polyphenol antioxidant (PA, ◊). Time 0 corresponds to pre-exercise baseline measurements. Measures at 5, 10, 15, and 20 min correspond to 40, 50, 60 and 70% of maximal oxygen consumption, respectively. From min 20 to 50 subjects cycled at 5% above their lactate threshold. A 10 min time trial started at min 50 and measurements were made every 2 min during this time trial. There were no significant differences between trials. * Indicates significant difference from previous value for PLAC and ^#^ indicates significant difference from previous value for PA, *p* < 0.05.

On day 1 the HR, SV, and CO responses during the first 50 min of exercise and 10 min TT were similar for PLAC and PA (Figure 4). MAP increased during exercise with no differences between treatments (Table 3). On day 2 there were no significant differences in HR, SV, or CO during for the two treatments (Table 4).

**Table 3:** Thermoregulatory and blood pressure responses; Day 1. Values are mean ± SD of 12 subjects.

Time, min	rest	5	10	15	20	50	52	54	56	58	60
**MAP, mmHg**											
**PLAC**	96 ± 5	108 ± 11	110 ± 10	110 ± 11	112 ± 10	114 ± 8	120 ± 7	124 ± 9	117 ± 10	122 ± 11	117 ± 10
**PA**	98 ± 6	106 ± 11	110 ± 11	110 ± 10	113 ± 9	116 ± 12	114 ± 10	118 ± 11	119 ± 10	116 ± 12	120 ± 11
**Tcore, °C**											
**PLAC**	37.3 ± 0.3	37.4 ± 0.3 *	37.5 ± 0.3 *	37.7 ± 0.3 *	37.8 ± 0.3 *	38.5 ± 0.3 *	38.5 ± 0.3 *	38.6 ± 0.3 *	38.7 ± 0.3 *	38.8 ± 0.3 *	38.9 ± 0.3 *
**PA**	37.3 ± 0.2	37.3 ± 0.2	37.4 ± 0.2 *	37.6 ± 0.2 *	37.8 ± 0.2 *	38.5 ± 0.3 *	38.6 ± 0.3 *	38.7 ± 0.3 *	38.7 ± 0.3 *	38.8 ± 0.3 *	38.9 ± 0.3 *
**Tskin, °C**											
**PLAC**	32.0 ± 0.9	32.2 ± 0.5	32.2 ± 0.5	32.3 ± 0.5	32.1 ± 0.7	31.0 ± 0.8 *	31.1 ± 0.8	31.1 ± 0.7	30.9 ± 0.7	31.0 ± 0.7	31.1 ± 1.0
**PA**	32.6 ± 0.7 ^†^	32.4 ± 0.6	32.2 ± 0.5	32.0 ± 0.4	32.1 ± 0.4	31.2 ± 1.0 *	31.1 ± 1.1	31.0 ± 1.1	30.9 ± 0.9	30.8 ± 1.0	30.6 ± 1.2
**SkBF, L/min**											
**PLAC**		1.3 ± 0.2	1.7 ± 0.3 *	2.0 ± 0.4 *	2.2 ± 0.4 *	1.7 ± 0.3 *					
**PA**		1.5 ± 0.2	1.7 ± 0.2 *	2.0 ± 0.2 *	2.2 ± 0.3 *	1.8 ± 0.3 *					

Placebo (PLAC), polyphenol antioxidant (PA), core temperature (Tcore), mean skin temperature (Tskin), mean arterial pressure (MAP), and skin blood flow (SkBF). * Indicates significant difference from previous value and ^†^ indicates significant difference from PLAC, *p* < 0.05.

**Table 4:** Cardiovascular and thermoregulatory responses during exercise at the lowest power output needed to elicit VO_2_max; Day 2. Values are mean ± SD of 12.

Time (% of completion)	0%	20%	40%	60%	80%	100%
**HR, bpm**						
**PLA**	154 ± 15	166 ± 13 *	173 ± 13 *	176 ± 13 *	179 ± 12 *	181 ± 12 *
**POM**	156 ± 14	166 ± 14 *	173 ± 13 *	176 ± 13 *	179 ± 12 *	181 ± 13
**SV, mL/beat**						
**PLA**	127 ± 13	129 ± 13	130 ± 16	130 ± 15	130 ± 15	130 ± 16
**POM**	130 ± 13	132 ± 17	133 ± 17	133 ± 15	133 ± 15	133 ± 17
**CO, L/min**						
**PLA**	19.6 ± 1.9	21.4 ± 2.0 *	22.4 ± 2.4 *	22.9 ± 2.2	23.2 ± 2.6	23.5 ± 2.9
**POM**	20.1 ± 1.9	21.8 ± 2.7 *	22.9 ± 2.7 *	23.4 ± 2.6 *	23.7 ± 2.6	24.0 ± 3.1
**Tcore, °C**						
**PLA**	37.7 ± 0.2	37.7 ± 0.2 *	37.8 ± 0.2 *	38.0 ± 0.2 *	38.1 ± 0.3 *	38.2 ± 0.3 *
**POM**	37.7 ± 0.2	37.8 ± 0.2	37.9 ± 0.2 *	38.0 ± 0.3 *	38.2 ± 0.3 *	38.3 ± 0.4 *
**Tskin, °C**						
**PLA**	32.3 ± 0.4	32.3 ± 0.4	32.1 ± 0.41 *	31.9 ± 0.42 *	31.9 ± 0.5	32.0 ± 0.4
**POM**	32.1 ± 0.7	32.2 ± 0.7	31.9 ± 0.7	31.9 ± 0.7	31.9 ± 0.7	31.7 ± 0.7

Placebo (PLAC), polyphenol antioxidant (PA), oxygen consumption (VO_2_), heart rate (HR), stroke volume (SV), cardiac output (CO), core temperature (Tcore), and skin temperature (Tskin). * Indicates significant difference from previous value, *p* < 0.05.

### 3.4. Thermoregulatory Responses

There were no pair-wise differences between treatments for Tcore, Tskin, or estimated SkBF during the one hour bout of exercise (Table 3). Tcore increased with each increase in power output during the first 20 min of exercise and continued to increase from min 20 to 50. During the 10 min TT, Tcore reached a peak value at the end of the TT (PLAC; 38.9 ± 0.3 °C and PA; 38.9 ± 0.3 °C) (Table 3). Tskin was slightly higher prior to exercise during the PA trials; however, by min 5 of exercise this difference was no longer present. Tskin was maintained between 32.0 °C and 32.3 °C from min 5 to min 20 and reduced by 1.1 °C (PLAC) and 0.8 °C (PA) from min 20 to min 50 (*p* < 0.05). Estimated SkBF increased with each increase in exercise intensity from min 5 to 20. At min 50, just prior to the initiation of the TT, SkBF was reduced compared to min 20. Overall, there were no pair-wise differences between trials at any time point during exercise for Tcore, Tskin, and SkBF.

Tcore and Tskin were not different between trials on day 2. Tcore at the start of the fatigue trial was 37.7 °C for both trials and increased to 38.3 °C. Tcore increased at a rate of approximately 0.1 °C/min. Tskin was maintained between 32.1 °C and 32.3 °C during the first 20% of the fatigue trial and then decreased by 0.4 °C until the final portion of the trial (Table 4).

## 4. Discussion

The primary finding of this study is that polyphenol antioxidant supplementation did not improve performance during prolonged exhaustive exercise (one hour of exercise including a 10 min time trial) or during shorter duration high intensity exercise (time to fatigue at VO_2_max) in the heat. Variables associated with performance including gross cycling efficiency, blood lactate accumulation, and maximal neuromuscular power in conjunction with cardiovascular and thermoregulatory responses to exercise were not altered by PA supplementation.

The importance of this study is highlighted by the recent rise in use and popularity of dietary antioxidant supplements for health and ergogenic purposes. Despite a rapid growth in popularity, scientific validation of the ergogenic benefits of antioxidants remains scarce. Antioxidants have been shown to improve [6,7,8,9,10,11,12], have no effect [13,14,15], or decrease [16] exercise performance. Similarly, adaptations associated with exercise have been shown to be improved [6], not changed [16], or decreased with antioxidant use [16,52,53,54]. In the current study, supplementation with a polyphenol antioxidant for 1 week failed to improve exercise performance, cardiovascular function, and thermoregulatory control in well-trained cyclists. The lack of improvement in exercise performance may be related to the training status of the subjects, training modality, and/or experimental conditions in which performance was assessed. Previous work from our laboratory, using nearly identical environmental and exercise conditions, reported significant improvements in performance due to carbohydrate and fluid ingestion in well trained cyclists [42] suggesting that the paradigm used in the current investigation was appropriate to identify improvements in performance due to nutritional interventions.

The rationale behind our choice of PA as our antioxidant supplement is based on finding that the high concentration of polyphenols, specifically ellagitannins (punicalagin and ellagic acid), found in pomegranates have been shown to possess higher antioxidant potency than other polyphenol rich fruits and beverages [28,29,30]. Pharmacokinetic analysis revealed that plasma levels of ellagic acid, a metabolite of the PA supplement, peaked 1 and 2 h post consumption and was present in some urine samples 48 h after initial ingestion [30]. Recent work from our lab [55,56] demonstrated that PA supplementation (identical composition as that used in the current study) was successful at attenuating the muscle weakness and soreness experienced following eccentric exercise that produced delayed onset muscle soreness. Given that subjects consumed the supplement twice daily for 7 days and consumed the supplement approximately 30 min before exercise we are confident that the active antioxidants from the supplement were in the system during exercise. Furthermore, the PA that we used has been shown to have high biological activity and potent treatment effects in clinical conditions associated with oxidative stress and inflammation such as type 2 diabetes [24], atherosclerosis [25], cancer [26], and rheumatoid arthritis [27]. Thus, it is unlikely that bioavailability of PA was a limiting factor leading to the absence of an ergogenic effect in the present study; however we cannot rule out the impact that a higher PA dose or longer supplementation period may have on endurance exercise performance. A limitation of the current study is the lack of blood markers of oxidative stress and antioxidant capacity following PA supplementation. However, the main focus of the investigation was to comprehensively assess exercise performance. Had performance been altered further mechanistic examination of antioxidant status would be warranted.

We chose to focus on cardiovascular and thermoregulatory function during exercise as PA has been shown to increase NO bioavailability by protecting NO from oxidative destruction [31]. Given that NO has important roles in cutaneous blood flow [32], thermoregulatory control of sweating during exercise [33], and skeletal muscle blood flow [34,35], we hypothesized that PA might reduce cardiovascular strain (*i.e*., HR, SV, CO) and improve thermoregulation during exercise in the heat. This was apparently not the case for our well-trained subjects as there were no differences in cardiovascular, thermoregulatory function, or requirements for skin blood flow between PLAC and PA.

Antioxidant administration has been shown to result in dramatic improvements in exercise performance and compelling evidence has been gathered showing the potential ergogenic properties of the antioxidant NAC [8,9,10,11,12,19]. Pertinent to the present study, a series of studies [8,10,11,12,57] documented significant improvements in time to fatigue during high intensity cycling. The mechanisms responsible for the improved performance may be associated with increased antioxidant availability [10] and improved K^+^ regulation as a result of maintained Na^+^/K^+^ pump [8,11]. Discrepancies between the aforementioned studies [8,10,11,12,57] and the present study may be due to a multitude of factors including: (1) method of administration (intravenous infusion *vs*. oral consumption); (2) the amount of antioxidant administered (physiological *vs*. supraphysiological); (3) the type of antioxidant provided (NAC *vs*. polyphenol); and (4) the environmental conditions under which exercise was performed (hot *vs*. temperate). While all of these differences may contribute to the disparate findings, the most likely explanations are the manner in which and the amount of the antioxidant that was administered. While the NAC intravenous infusion studies [8,10,11,12,57] provide valuable information regarding mechanisms related to muscle fatigue and the role of reactive oxygen species (ROS) and free radicals in muscle fatigue, they do not, from a practical and application oriented perspective, provide information regarding the ergogenic benefit of oral antioxidant supplementation.

There are a limited number of investigations using physiological amounts of oral antioxidant supplements and the results regarding exercise performance are equivocal. The only human study [7], to our knowledge, to report a significant improvement in exercise performance found a small but significant 3% increase in 30 km time trial performance after 6 weeks of quercetin supplementation. Similarly, Davis *et al*. [6] fed mice quercetin for 7 days and showed improved exercise tolerance (time to fatigue on a treadmill) and mitochondrial biogenesis. In support of the findings of the present study, Cheuvront *et al*. [13] reported that quercetin supplementation (2000 mg) did not affect total work performed during a 15 min time trial in the heat or have any impact on the on physiological or perceptual measures during exercise. Neiman *et al*. [14] found no difference between quercetin and placebo in any performance related measurement during consecutive days of cycling. These previous findings [13,14], coupled with the results of the present study, suggest that antioxidant supplementation, in general, does not improve exercise performance in healthy endurance-trained cyclists.

The present study was designed in order to increase the oxidative stress that is associated with exercise by performing high intensity exercise with the addition of a heat stress. Both high intensity exercise and heat stress have been shown to induce an excessive elevation in free radical production and subsequent increase in oxidative stress beyond normal conditions [5,58]. Free radical production appears to play a necessary or regulatory role in many physiological processes and functions including mitochondrial biogenesis, vascular function, and inflammation. Recent findings argue that antioxidant supplementation in healthy individuals may disrupt and have a negative impact on physiological function, thereby attenuating or even reducing the positive outcomes associated with exercise and exercise training [16,53,54]. Despite the finding of no effect of antioxidant supplementation in our healthy well-trained subjects during endurance exercise, a similar conclusion that antioxidant supplementation is unnecessary, or not beneficial, cannot be generalized to all situations in which oxidative stress is acutely or chronically elevated. Healthy individuals subjected to elevated levels of oxidative stress due to eccentric exercise producing muscle damage, weakness [55,56] or inflammation [59,60] exhibit significant improvements in recovery following antioxidant supplementation. Similarly antioxidant supplementation may be warranted in situations in which oxidative stress is chronically elevated (aging, disease, and dysfunction) [24,25,26,27,61,62,63,64]. Under these conditions exogenous antioxidants appear to compensate for the inability of the endogenous antioxidant systems to combat the chronic increase in oxidative stress.

## 5. Conclusions

Based on the findings of this study, polyphenol antioxidant supplementation had no beneficial effect on performance during both prolonged and short duration exhaustive exercise in the heat in endurance-trained cyclists. Accordingly, polyphenol antioxidant supplementation had no effect on gross cycling efficiency, rating of perceived exertion, or the cardiovascular and thermoregulatory responses to exercise. Overall, these findings question the use of polyphenol antioxidant supplementation as an ergogenic aid aimed at improving endurance exercise performance.

## References

[1] Davies K., Quintanilha A., Brooks G., Packer L. (1982). Free radicals and tissue damage produced by exercise. Biochem. Biophys. Res. Commun..

[2] Powers S.K., Jackson M.J. (2008). Exercise-Induced Oxidative Stress: Cellular Mechanisms and Impact on Muscle Force Production. Physiol. Rev..

[3] Bailey D.M., Young I.S., McEneny J., Lawrenson L., Kim J., Barden J., Richardson R.S. (2004). Regulation of free radical outflow from an isolated muscle bed in exercising humans. Am. J. Physiol. Heart Circ. Physiol..

[4] Flanagan S., Moseley P., Buettner G. (1998). Increased flux of free radicals in cells subjected to hyperthermia: Detection by electron paramagnetic resonance spin trapping. FEBS Lett..

[5] McAnulty S., McAnulty L., Pascoe D., Gropper S., Keith R., Morrow J., Gladden L. (2005). Hyperthermia increases exercise-induced oxidative stress. Int. J. Sports Med..

[6] Davis J.M., Murphy E.A., Carmichael M.D., Davis B. (2009). Quercetin increases brain and muscle mitochondrial biogenesis and exercise tolerance. Am. J. Physiol. Regul. Integr. Comp. Physiol..

[7] MacRae H., Mefferd K. (2006). Dietary antioxidant supplementation combined with quercetin improves cycling time trial performance. Int. J. Sport Nutr. Exerc. Metab..

[8] McKenna M.J., Medved I., Goodman C.A., Brown M.J., Bjorksten A.R., Murphy K.T., Petersen A.C., Sostaric S., Gong X. (2006). *N*-Acetylcysteine attenuates the decline in muscle Na^+^, K^+^-pump activity and delays fatigue during prolonged exercise in humans. J. Physiol..

[9] McKenna M.J., Bangsbo J., Renaud J.-M. (2008). Muscle K^+^, Na^+^, and Cl^−^ disturbances and Na^+^-K^+^ pump inactivation: Implications for fatigue. J. Appl. Physiol..

[10] Medved I., Brown M.J., Bjorksten A.R., Murphy K.T., Petersen A.C., Sostaric S., Gong X., McKenna M.J. (2004). *N*-Acetylcysteine enhances muscle cysteine and glutathione availability and attenuates fatigue during prolonged exercise in endurance-trained individuals. J. Appl. Physiol..

[11] Medved I., Brown M.J., Bjorksten A.R., McKenna M.J. (2004). Effects of intravenous *N*-acetylcysteine infusion on time to fatigue and potassium regulation during prolonged cycling exercise. J. Appl. Physiol..

[12] McKenna M.J., Hargreaves M. (2008). Resolving fatigue mechanisms determining exercise performance: Integrative physiology at its finest!. J. Appl. Physiol..

[13] Cheuvront S.N., Ely B.R., Kenefick R.W., Michniak-Kohn B.B., Rood J.C., Sawka M.N. (2009). No effect of nutritional adenosine receptor antagonists on exercise performance in the heat. Am. J. Physiol. Regul. Integr. Comp. Physiol..

[14] Nieman D., Henson D., Maxwell K., Williams A., McAnulty S., Jin F., Shanely R., Lines T. (2009). Effects of quercetin and EGCG on mitochondrial biogenesis and immunity. Med. Sci. Sports Exerc..

[15] Utter A., Nieman D., Kang J., Dumke C., Quindry J., McAnulty S., McAnulty L. (2009). Quercetin does not affect rating of perceived exertion in athletes during the Western States endurance run. Res. Sports Med..

[16] Gomez-Cabrera M.-C., Domenech E., Romagnoli M., Arduini A., Borras C., Pallardo F.V., Sastre J., Vina J. (2008). Oral administration of vitamin C decreases muscle mitochondrial biogenesis and hampers training-induced adaptations in endurance performance. Am. J. Clin. Nutr..

[17] Medved I., Brown M.J., Bjorksten A.R., Leppik J.A., Sostaric S., McKenna M.J. (2003). *N*-Acetylcysteine infusion alters blood redox status but not time to fatigue during intense exercise in humans. J. Appl. Physiol..

[18] Traveline J.M., Sudarshan S., Roy B.G., Cordova F., Leyenson V., Criner G.J. (1997). Effect of *N*-Acetylcysteine on human diaphragm strength and fatigability. Am. J. Respir. Crit. Care Med..

[19] Matuszczak Y., Farid M., Jones J., Lansdowne S., Smith M., Taylor A., Reid M. (2005). Effects of *N*-acetylcysteine on glutathione oxidation and fatigue during handgrip exercise. Muscle Nerve.

[20] Hernandez A., Cheng A., Westerblad H. (2012). Antioxidants and Skeletal Muscle Performance: “Common Knowledge” *vs*. Experimental Evidence. Front Physiol..

[21] Seeram N.P., Adams L.S., Henning S.M., Niu Y., Zhang Y., Nair M.G., Heber D. (2005). *In vitro* antiproliferative, apoptotic and antioxidant activities of punicalagin, ellagic acid and a total pomegranate tannin extract are enhanced in combination with other polyphenols as found in pomegranate juice. J. Nutr. Biochem..

[22] Scalbert A., Johnson I.T., Saltmarsh M. (2005). Polyphenols: Antioxidants and beyond. Am. J. Clin. Nutr..

[23] Vita J.A. (2005). Polyphenols and cardiovascular disease: Effects on endothelial and platelet function. Am. J. Clin. Nutr..

[24] Rosenblat M., Hayek T., Aviram M. (2006). Anti-oxidative effects of pomegranate juice (PJ) consumption by diabetic patients on serum and on macrophages. Atherosclerosis.

[25] Aviram M., Rosenblat M., Gaitini D., Nitecki S., Hoffman A., Dornfeld L., Volkova N., Presser D., Attias J., Liker H. (2004). Pomegranate juice consumption for 3 years by patients with carotid artery stenosis reduces common carotid intima-media thickness, blood pressure and LDL oxidation. Clin. Nutr..

[26] Adams L., Seeram N., Aggarwal B., Takada Y., Sand D., Heber D. (2006). Pomegranate juice, total pomegranate ellagitannins, and punicalagin suppress inflammatory cell signaling in colon cancer cells. J. Agric. Food Chem..

[27] Shukla M., Gupta K., Rasheed Z., Khan K., Haqqi T. (2008). Consumption of hydrolyzable tannins-rich pomegranate extract suppresses inflammation and joint damage in rheumatoid arthritis. Nutrition.

[28] Seeram N., Aviram M., Zhang Y., Henning S., Feng L., Dreher M., Heber D. (2008). Comparison of antioxidant potency of commonly consumed polyphenol-rich beverages in the United States. J. Agric. Food Chem..

[29] Seeram N., Zhang Y., McKeever R., Henning S., Lee R., Suchard M., Li Z., Chen S., Thames G., Zerlin A. (2008). Pomegranate juice and extracts provide similar levels of plasma and urinary ellagitannin metabolites in human subjects. J. Med. Food.

[30] Seeram N.P., Henning S.M., Zhang Y., Suchard M., Li Z., Heber D. (2006). Pomegranate Juice Ellagitannin Metabolites Are Present in Human Plasma and Some Persist in Urine for Up to 48 h. J. Nutr..

[31] Ignarro L., Byrns R., Sumi D., de Nigris F., Napoli C. (2006). Pomegranate juice protects nitric oxide against oxidative destruction and enhances the biological actions of nitric oxide. Nitric Oxide.

[32] Kellogg D.L., Crandall C.G., Liu Y., Charkoudian N., Johnson J.M. (1998). Nitric oxide and cutaneous active vasodilation during heat stress in humans. J. Appl. Physiol..

[33] Welch G., Foote K.M., Hansen C., Mack G.W. (2009). Nonselective NOS inhibition blunts the sweat response to exercise in a warm environment. J. Appl. Physiol..

[34] Boushel R., Langberg H., Gemmer C., Olesen J., Crameri R., Scheede C., Sander M., Kjaer M. (2002). Combined inhibition of nitric oxide and prostaglandins reduces human skeletal muscle blood flow during exercise. J. Physiol..

[35] Mortensen S.P., Gonzalez-Alonso J., Damsgaard R., Saltin B., Hellsten Y. (2007). Inhibition of nitric oxide and prostaglandins, but not endothelial-derived hyperpolarizing factors, reduces blood flow and aerobic energy turnover in the exercising human leg. J. Physiol..

[36] Bailey S.J., Fulford J., Vanhatalo A., Winyard P.G., Blackwell J.R., DiMenna F.J., Wilkerson D.P., Benjamin N., Jones A.M. (2010). Dietary nitrate supplementation enhances muscle contractile efficiency during knee-extensor exercise in humans. J. Appl. Physiol..

[37] Bailey S.J., Winyard P., Vanhatalo A., Blackwell J.R., DiMenna F.J., Wilkerson D.P., Tarr J., Benjamin N., Jones A.M. (2009). Dietary nitrate supplementation reduces the O2 cost of low-intensity exercise and enhances tolerance to high-intensity exercise in humans. J. Appl. Physiol..

[38] Jones A.M., Bailey S.J., Vanhatalo A. (2013). Dietary Nitrate and O_2_ Consumption during Exercise. Med. Sport Sci..

[39] Larsen F.J., Schiffer T.A., Borniquel S., Sahlin K., Ekblom B., Lundberg J.O., Weitzberg E. (2011). Dietary inorganic nitrate improves mitochondrial efficiency in humans. Cell Metab..

[40] Martin J., Wagner B., Coyle E. (1997). Inertial-load method determines maximal cycling power in a single exercise bout. Med. Sci. Sports Exerc..

[41] Coyle E., Sidossis L., Horowitz J., Beltz J. (1992). Cycling efficiency is related to the percentage of type I muscle fibers. Med. Sci. Sports Exerc..

[42] Below P., Mora-Rodriguez R., Gonzalez-Alonso J., Coyle E. (1995). Fluid and carbohydrate ingestion independently improve performance during 1 h of intense exercise. Med. Sci. Sports Exerc..

[43] Trinity J.D., Lee J.F., Pahnke M.D., Beck K.C., Coyle E.F. (2012). Attenuated relationship between cardiac output and oxygen uptake during high-intensity exercise. Acta Physiol..

[44] Dingwell J., Joubert J., Diefenthaeler F., Trinity J. (2008). Changes in muscle activity and kinematics of highly trained cyclists during fatigue. IEEE Trans. Biomed. Eng..

[45] Charloux A., Lonsdorfer-Wolf E., Richard R., Lampert E., Oswald-Mammosser M., Mettauer B., Geny B., Lonsdorfer J. (2000). A new impedance cardiograph device for the non-invasive evaluation of cardiac output at rest and during exercise: Comparison with the “direct” Fick method. Eur. J. Appl. Physiol..

[46] Richard R., Lonsdorfer-Wolf E., Charloux A., Doutreleau S., Buchheit M., Oswald-Mammosser M., Lampert E., Mettauer B., Geny B., Lonsdorfer J. (2001). Non-invasive cardiac output evaluation during a maximal progressive exercise test, using a new impedance cardiograph device. Eur. J. Appl. Physiol..

[47] Hardy J.D., Du Bois E.F., Soderstrom G.F. (1938). The Technic of Measuring Radiation and Convection: One Figure. J. Nutr..

[48] Borg G., Borg G. (1975). Simple Rating Methods for Estimation of Perceived Exertion. Physical Work and Effort.

[49] Sawka M.N., Wenger C., Pandolf K.B., Sawka M.N., Gonzalez R.R. (1988). Physiological Responses to Acute Exercise-heat Stress. Human Performance Physiology and Environmental Medicine at Terrestrial Extremes.

[50] Trinity J., Pahnke M., Reese E., Coyle E. (2006). Maximal mechanical power during a taper in elite swimmers. Med. Sci. Sports Exerc..

[51] Trinity J., Pahnke M., Sterkel J., Coyle E. (2008). Maximal power and performance during a swim taper. Int. J. Sports Med..

[52] Lescaudron L., Peltekian E., Fontaine-Perus J., Paulin D., Zampieri M., Garcia L., Parrish E. (1999). Blood borne macrophages are essential for the triggering of muscle regeneration following muscle transplant. Neuromuscul. Disord..

[53] Richardson R.S., Donato A.J., Uberoi A., Wray D.W., Lawrenson L., Nishiyama S., Bailey D.M. (2007). Exercise-induced brachial artery vasodilation: Role of free radicals. Am. J. Physiol. Heart Circ. Physiol..

[54] Wray D., Uberoi A., Lawrenson L., Bailey D., Richardson R. (2009). Oral antioxidants and cardiovascular health in the exercise-trained and untrained elderly: A radically different outcome. Clin. Sci..

[55] Trombold J.R., Reinfeld A.S., Casler J.R., Coyle E.F. (2011). The effect of pomegranate juice supplementation on strength and soreness after eccentric exercise. J. Strength Cond. Res..

[56] Trombold J.R., Barnes J.N., Critchley L., Coyle E.F. (2010). Ellagitannin comsumption improves strength recovery 2–3 days following eccentric exercise. Med. Sci. Sports Exerc..

[57] Murphy K.T., Medved I., Brown M.J., Cameron-Smith D., McKenna M.J. (2008). Antioxidant treatment with *N*-acetylcysteine regulates mammalian skeletal muscle Na^+^-K^+^-ATPaseα gene expression during repeated contractions. Exp. Physiol..

[58] Tyldum G.A., Schjerve I.E., Tjonna A.E., Kirkeby-Garstad I., Stolen T.O., Richardson R.S., Wisloff U. (2009). Endothelial Dysfunction Induced by Post-Prandial Lipemia: Complete Protection Afforded by High-Intensity Aerobic Interval Exercise. J. Am. Coll. Cardiol..

[59] Plotnick G.D., Corretti M.C., Vogel R.A., Hesslink R., Wise J.A. (2003). Effect of supplemental phytonutrients on impairment of the flow-mediated brachialartery vasoactivity after a single high-fat meal. J. Am. Coll. Cardiol..

[60] Barringer T.A., Hatcher L., Sasser H.C. (2008). Potential Benefits on Impairment of Endothelial Function after a High-fat Meal of 4 weeks of Flavonoid Supplementation. Evid. Based Complement. Altern. Med..

[61] Eskurza I., Monahan K.D., Robinson J.A., Seals D.R. (2004). Effect of acute and chronic ascorbic acid on flow-mediated dilatation with sedentary and physically active human ageing. J. Physiol..

[62] Kirby B.S., Voyles W.F., Simpson C.B., Carlson R.E., Schrage W.G., Dinenno F.A. (2009). Endothelium-dependent vasodilatation and exercise hyperaemia in ageing humans: Impact of acute ascorbic acid administration. J. Physiol..

[63] Jablonski K.L., Seals D.R., Eskurza I., Monahan K.D., Donato A.J. (2007). High-dose ascorbic acid infusion abolishes chronic vasoconstriction and restores resting leg blood flow in healthy older men. J. Appl. Physiol..

[64] Donato A.J., Uberoi A., Bailey D.M., Walter Wray D., Richardson R.S. (2010). Exercise-induced brachial artery vasodilation: Effects of antioxidants and exercise training in elderly men. Am. J. Physiol. Heart Circ. Physiol..

